# Low prevalence of blood parasites in a long-distance migratory raptor: the importance of host habitat

**DOI:** 10.1186/s13071-015-0802-9

**Published:** 2015-03-31

**Authors:** Rafael Gutiérrez-López, Laura Gangoso, Josué Martínez-de la Puente, Jakob Fric, Pascual López-López, Mélanie Mailleux, Joaquín Muñoz, Laïd Touati, Boudjema Samraoui, Jordi Figuerola

**Affiliations:** Estación Biológica de Doñana (EBD-CSIC), C/Américo Vespucio, s/n, E-41092 Seville, Spain; Hellenic Ornithological Society, Themistokleous str. 80, 10681 Athens, Greece; Vertebrates Zoology Research Group, University of Alicante, E-03080 Alicante, Spain; Biology and Ecology Department, University of Constantine, 25017 Constantine, Algeria; Laboratoire de Recherche et de Conservation des Zones Humides, University of Guelma, 24000 Guelma, Algeria; Centre of Excellence for Research in Biodiversity, King Saud University, 12643 Riyadh, Saudi Arabia

**Keywords:** *Plasmodium*, *Haemoproteus*, *Leucocytozoon*, Eleonora’s falcon, Marine habitats, Migratory species, Vectors

## Abstract

**Background:**

The low prevalence of blood parasites in some bird species may be related to the habitats they frequent, the inexistence of the right host-parasite assemblage or the immunological capacity of the host. Here, we assess the parasite load of breeding populations of Eleonora’s falcon (*Falco eleonorae*), a medium-sized long-distance migratory raptor that breeds on small isolated islets throughout the Mediterranean basin and overwinters in inland Madagascar.

**Methods:**

We examined the prevalence and genetic diversity of the blood parasites belonging to the genera *Plasmodium*, *Haemoproteus* and *Leucocytozoon* in Eleonora’s falcon nestlings from five colonies and in adults from two colonies from nesting sites distributed throughout most of the species’ breeding range.

**Results:**

None of the 282 nestlings analysed were infected by blood parasites; on the other hand, the lineages of *Plasmodium*, *Haemoproteus* and *Leucocytozoon* were all found to infect adults. Our results support the idea of no local transmission of vector-borne parasites in marine habitats. Adult Eleonora’s falcons thus may be infected by parasites when on migration or in their wintering areas.

**Conclusion:**

The characteristics of marine environments with a lack of appropriate vectors may thus be the key factor determining the absence of local transmission of blood parasites. By comparing the parasite lineages isolated in this species with those previously found in other birds we were able to infer the most likely areas for the transmission of the various parasite lineages.

## Background

The presence and abundance of insect vectors is a key factor affecting the interaction between blood parasites and wild bird populations [1,2]. Indeed, habitat characteristics influence both birds’ habitat choice during the breeding season and the viability of insect vector populations, and may ultimately determine the success of blood parasite transmission [3]. Piersma [4] has suggested that bird species inhabiting marine habitats such as small isolated islets or sea cliffs usually have lower blood parasite prevalence than species inhabiting inland areas (*i.e*. mainland and/or large islands) due to the scarcity of insect vectors in marine habitats. Marine habitats whose environments are characterized by high salinity, exposure to winds and a lack of vegetation cover are generally unsuitable places for insect vectors that require an aquatic larval stage to complete their life-cycles [5]. In fact, a number of studies on seabirds have found a low prevalence or total absence of blood parasites and the suggested cause is the scarcity of insect vectors [4,6,7]. However, in addition to the role of vectors, other factors such as the existence of the right host-parasite assemblage and/or the immunological capacity of the avian host to fight off infections may also affect the outcome of host-blood parasite interactions [3,8]. Migratory bird species that use a range of habitats throughout their life-cycles (e.g. marine and freshwater inland habitats) are excellent study models for exploring the relative importance of the potential mechanisms involved in parasite transmission.

In this study, we assessed variation in blood parasite prevalence between breeding colonies and host status (nestlings *vs.* breeding adults) in Eleonora’s falcon (*Falco eleonorae*). This long-distance migratory raptor breeds colonially on small isolated islets throughout the Mediterranean basin and overwinters in inland Madagascar [9-11]. We used a PCR-based approach to determine the prevalence and the genetic identity of three avian blood parasite genera, *Plasmodium, Haemoproteus* and *Leucocytozoon*, that potentially infect these falcons. These parasite genera commonly infect birds and all have similar life cycles that require the presence of haematophagous insect vectors if they are to be transmitted [12].

**Figure 1:**
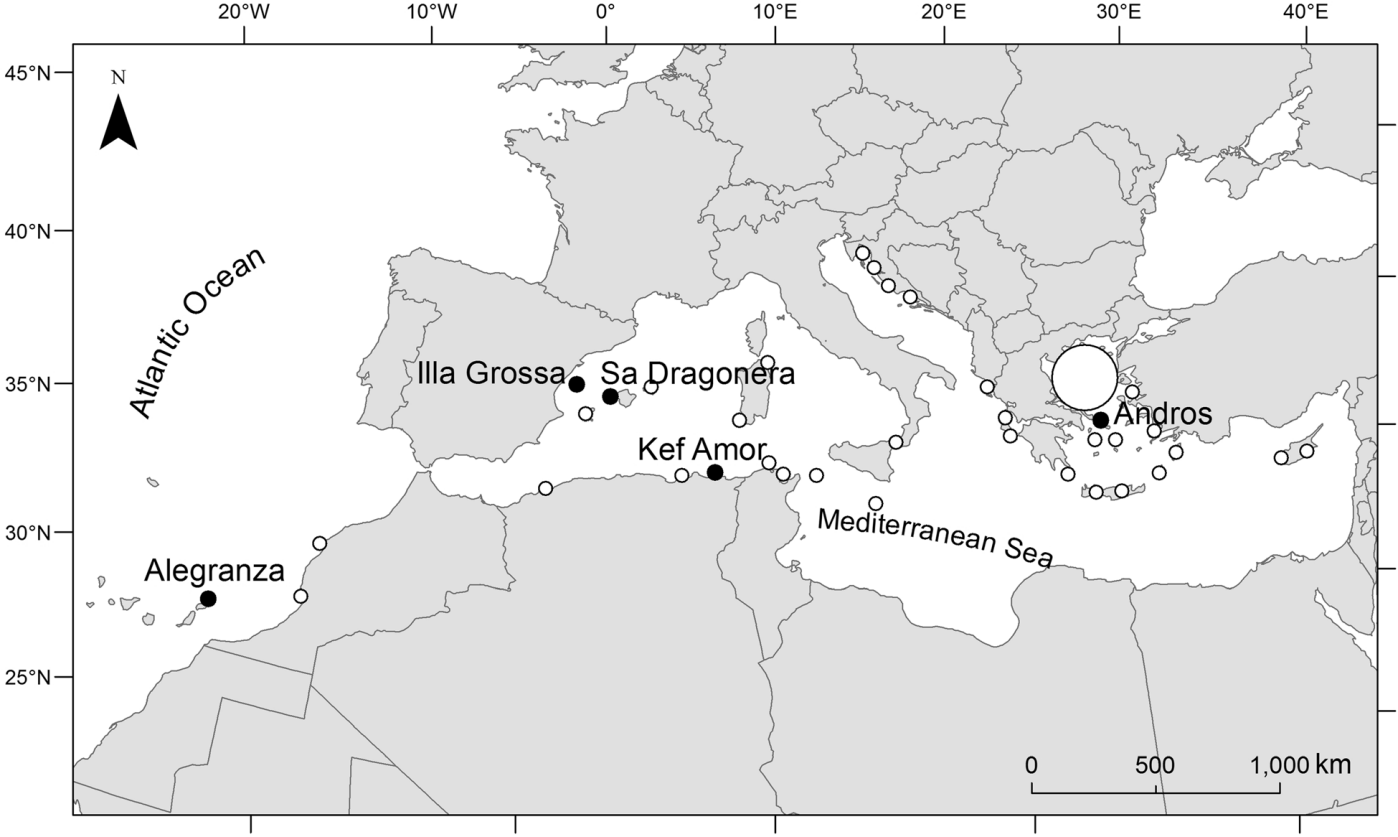
**Map of the entire breeding range of Eleonora’s falcon.** Solid black circles show the colonies where blood sampling was carried out for this study. The other colonies of the species are represented by white circles.

In the light of Piersma’s hypothesis [4], we expected to find a general paucity of blood parasites in Eleonora’s falcons in marine habitats. To determine the relative importance of habitat-related *vs*. host-related mechanisms on parasite transmission, we 1) compared the parasite load in nestlings from five different breeding colonies located in sites scattered throughout most of the species’ breeding range (Figure 1) to determine whether or not local transmission of vector-borne blood parasites occurs on marine breeding grounds, and 2) compared the blood parasite prevalence in nestlings with that of adults from two of these breeding areas. Unlike nestlings, adult Eleonora’s falcons are exposed during their annual cycle to a huge range of habitats and their associated pathogens.

## Methods

### The vertebrate host

Eleonora’s falcon breeds colonially in marine environments, mainly on the sea cliffs of small islands and rocky islets in the Mediterranean Basin (from Spain to Greece), as well as on several islets in the eastern Atlantic Ocean (the Canary archipelago and the Îles Purpuraires off the north coast of Africa) [9]. When raising offspring, this species is highly specialized in the hunting of migratory birds that are heading to Africa. Accordingly, breeding colonies are strategically situated on small islands and islets located along the main migratory flyways. This falcon has a delayed breeding season and is the tardiest breeder (August–October) of all Northern Hemisphere raptor species [13]. They lay a single clutch of 1–4 eggs. Incubation lasts for 31 ± 2 days and nestlings fledge at 35–40 days [14]. After breeding, Eleonora’s falcons undertake a long-distance migration across continental Africa to their winter quarters in Madagascar [11]. This migratory journey takes about 1–2 months and passes through at least 12 countries where they perform several stopovers [11]. In winter, Eleonora’s falcons shift both their choice of food items (from birds to insects) and habitat and occupy humid areas of northern-central Madagascar, where the high rainfall can lead to an abundance of insects [15].

### Study area and blood sampling

We sampled five Eleonora’s falcon populations located in three different countries in the Mediterranean Basin (Figure 1) in 2008–2012. These populations were selected to cover most of the species’ breeding range, from the westernmost (Alegranza Islet, Canary Islands, Spain) to the easternmost (Andros islet, Cyclades Islands, Greece) breeding sites (Figure 1). All birds were measured, bled and released after handling. Wing length (mm) was used to estimate nestling age (±1 day) as per Ristow and Wink [16]. When nestlings were 25–28 days old, a blood sample (0.2 ml) was extracted with a syringe from the brachial vein. In a previous study, it was found that 16–23-days old sparrowhawk (*Accipiter nisus*) nestlings were infected by *Haemoproteus* and *Leucozytozoon* [17]. This supports the idea 25 days is enough time for blood parasites to be detected in the peripheral blood of Eleonora’s falcon nestlings. In addition, the adult Eleonora’s falcons, i.e., individuals that had performed at least one complete migration and had overwintered in inland Madagascar, from both Illa Grossa and Alegranza, were trapped using dho-gaza nets and a stuffed eagle owl (*Bubo bubo*) as a decoy. Adult birds were bled in the same way as nestlings. Blood samples were preserved in absolute ethanol and stored at −20°C until molecular analysis.

### DNA extraction and blood parasite analyses

Genomic DNA was isolated from blood samples using a standard chloroform/isoamyl alcohol method [18]. A 478 bp fragment of the mitochondrial cytochrome *b* gene of blood parasites was amplified as per Hellgren *et al.* [19]. The presence of amplicons was verified in 1.8% agarose gels. All negative samples in a first screening were repeated twice to minimize the possibility of false negatives. Positive samples obtained using the standard chloroform/isoamyl alcohol method were re-extracted using the Qiagen DNeasy® Kit Tissue and Blood (Qiagen, Hilden, Germany) and a further PCR was performed to identify blood parasites lineages. We used this second step for sequencing because the quality of DNA sequences – but not the amplification success – significantly improves using this commercial method when compared to the standard chloroform/isoamyl alcohol method [20]. Sequencing reactions were performed according using the BigDye technology (Applied Biosystems) and sequenced in both directions through a 3130xl ABI automated sequencer (Applied Biosystems). The primers HaemF and HaemR2 for *Plasmodium* and *Haemoproteus* genera and HaemFL and HaemR2L for *Leucocytozoon* genus were used. Sequences were edited using the SequencherTM v4.9 software (Gene Codes Corp., © 1991–2009, Ann Arbor, MI 48108). Parasite lineages were identified by comparison with sequences deposited in GenBank (National Center for Biotechnology Information, Blast, 2008). Blood parasite prevalences in adults and nestlings from Alegranza, the only locality where blood parasites were detected (see results), were compared using Chi-square tests (Statistica V. 7.0, StatSoft, I.N.C. 2001).

### Ethical approval details

Corresponding permissions were issued by the Spanish, Algerian, and Greek Regional Administrations, according to National laws. Specific permissions numbers: MAOT N° 11908, MAOT N° 6468, MAOT N° 9723, E-87-10-T, E-59-11-E, and 95144/42.

## Results

Out of the total of 324 individuals sampled (282 nestlings and 42 adults, see Table 1), blood parasite infections were only found in seven adult falcons (7/42; prevalence in adults = 16.7%), all from the Alegranza population (adult intrapopulation prevalence = 20.0%). None of the nestlings analysed had blood parasites. Parasite infection differed significantly between age classes in Alegranza, the only population where infections were detected (adults: 7/35, nestlings: 0/173; χ^2^ = 29.92, d. f. = 1, P < 0.0001).

**Table 1:** **Summary of the Eleonora’s falcon breeding populations and sample sizes used in this study**

**Age**	**Locality**	**Infected/Sampled**	**Parasite lineages (number of infected birds)**
Nestlings			
	Alegranza	0/173	
	Illa Grossa	0/36	
	Sa Dragonera	0/11	
	Kef Amor	0/44	
	Andros islet	0/18	
Adults			
	Alegranza	7/35	*Haemoproteus* LK4 (3), *Haemoproteus* hBUBIBI01 (1), *Plasmodium* LK6 (2), *Leucocytozoon* L_CIAE02 (1)
	Illa Grossa	0/7	

The number of infected hosts and the parasite identity are also shown.

Overall, we found four different genetic lineages infecting adult Eleonora’s falcons: two *Haemoproteus* lineages (lineage *LK4,* which was isolated from three adults, and lineage *hBUBIBI01,* which was isolated from a single individual); *Plasmodium* lineage *LK6* (isolated from two adults); and a single individual infected by *Leucocytozoon* lineage *L_CIAE02*. None of the adults showed any evidence of infection by more than one parasite lineage.

## Discussion

We found that none of the Eleonora’s falcon nestlings from any of the populations in the Mediterranean basin was infected by blood parasites; on the other hand, 20.0% of the adults from the Alegranza population were infected by at least one of the blood parasite genera identified (Table 1). However, the overall prevalence of each blood parasite lineage infecting the adults in this population was very low, ranging from 2.86% (*Leucocytozoon* L_CIAE02) to 8.57% (*Haemoproteus* LK4). The absence of parasite infections in adults from Illa Grossa and from nestlings from Sa Dragonera could be due to the low sample sizes, which may have biased our estimations of blood parasite prevalence in these two populations [21]. Even so, results from the other populations suggest a complete absence of infection by blood parasites in nestlings. In a previous study, Gangoso *et al.* [22] reported the absence of antibodies against the mosquito-borne West Nile virus in Eleonora’s falcon nestlings from Alegranza, despite being detected in 14.8% of adults from the same population. This finding agrees with the results of our study regarding different vector-borne pathogens. In addition, Martínez-Abraín and Urios [23] found no blood parasites infecting Eleonora’s falcon nestlings from the Columbretes Islands. Nevertheless, Wink *et al.* [24] found that 18.8% of adult Eleonora’s falcons breeding in the Aegean Sea were infected by *Leucocytozoon*; regretfully, these authors provide no information about nestlings. Unlike our study, Martínez-Abraín and Urios [23] and Wink *et al*. [24] used blood smears for parasite detection. Nonetheless, our findings, in which a molecular approach was used, agree with the results of these authors’ studies.

Although information regarding the development of blood parasites in nestlings of wild bird populations is scarce, studies conducted in different avian groups have detected avian blood parasites infecting nestlings after as few as 13 days of life [2,25-27]. Svobodová *et al*. [17] found *Haemoproteus* and *Leucocytozoon* in 16–23-days old sparrowhawk (*Accipiter nisus*) nestlings. Therefore, it is unlikely that the absence of parasites in Eleonora’s falcon nestlings was due to time constraints in parasite development.

The presence of appropriate insect vectors is a crucial factor influencing the success of blood parasite transmission in birds [1,2]. Mosquitoes (Fam. Culicidae), biting midges (Fam. Ceratopogonidae) and black flies (Fam. Simuliidae) are the main vectors of *Plasmodium*, *Haemoproteus* and *Leucocytozoon*, respectively [12]. Although we did not perform any entomological surveillance to quantify insect diversity and abundance in the study areas, no previous study has ever found any of these insect vectors on either Alegranza [22] or the Columbretes Islands [6]. Eleonora’s falcons are usually parasitized by blood-sucking louse flies (Hippoboscidae) [22,24], which, in the absence of other vectors, could play a role in the transmission of blood parasites in marine habitats. Indeed, a recent study reported that *Hemoproteus iwa* in Galapagos great frigatebirds (*Fregata minor*) was vectored by a hippoboscid fly, an obligate ectoparasite of the bird host [28]. However, louse flies can transmit parasites of the subgenus *Haemoproteus*, as is the case of *H. iwa* [12], but not of the subgenus *Parahaemoproteus,* which were isolated from the adult Eleonora’s falcons in this study. In addition, the subgenus *Haemoproteus* seems to be restricted to pigeons and frigatebirds [29,30] and, to our knowledge, have not been found to infect falcons. Therefore, the lack of suitable vectors might explain the incapacity of transmission from infected adults to uninfected nestlings in breeding areas.

Alternatively, the lack of blood parasites in Eleonora’s falcon nestlings in these five populations could be due to host-related immune mechanisms, as suggested by Martínez-Abraín *et al*. [8]. However, this possibility is poorly supported by our results, since the prevalence of infection in adults found in this and previous studies [24] showed that Eleonora’s falcons are indeed susceptible to blood parasite infections. The Eleonora’s falcon possesses a very specialized Major Histocompatibility Complex (MHC), characterized by a complete lack of variability at both MHC class I and II, probably due to pathogen-driven selection [31]. The MHC system may play a key role in the defence of birds against blood parasites [32]. Further studies should be conducted to identify the role of the specialized MHC system in the mechanisms used by Eleonora’s falcon against blood parasite infections.

We suggest that either during migration or in wintering areas, adult Eleonora’s falcons may encounter a diversity of vectors transmitting *Plasmodium*, *Haemoproteus* and *Leucocytozoon*. After breeding, Eleonora’s falcons perform a long-distance migration across continental Africa to reach Madagascar [10,33], thus crossing and stopping in areas with a high abundance of potential insect vectors during the rainy season [33,34]. Njabo *et al*. [35] and Waldenström *et al*. [36] isolated *Plasmodium* and *Haemoproteus* parasites in wild mosquitoes from Cameroon and in migratory and resident birds from Nigeria, respectively. In addition, *Haemoproteus* (17.4% prevalence), *Leucocytozoon* (9.4%) and *Plasmodium* (1.9%) have been found in birds from different families in Madagascar [37]. In their wintering quarters, Eleonora’s falcons inhabit degraded humid forests and cultivated areas close to pristine humid forest [15] where, due to their suitability for insect vector reproduction, blood parasite transmission may occur. Furthermore, during the pre-breeding and breeding seasons, adult Eleonora’s falcons often travel inland (i.e. the main islands of the Canary and Balearic archipelagos, continental Africa and continental Europe) to visit freshwater ponds and other water bodies [38], where the presence of suitable vectors such as biting midges [39] and mosquitoes [40] has been recorded.

By comparing the parasite lineages isolated from Eleonora’s falcons with those previously found in other wild bird species, it is possible to infer areas of parasite transmission and determine the host-range of these parasite lineages. In this respect, *Plasmodium* LK6 and *Haemoproteus* LK4 lineages have been isolated from adults of the closely related lesser kestrel (*Falco naumannii*) in Spain [41,42], with a parasite prevalence of 4.6% and 0.7% in adults, respectively. Like Eleonora’s falcon, the lesser kestrel is a long-distance migratory species that winters in Africa, which suggests that parasite-vector interactions in wintering quarters may facilitate the transmission of blood parasites in these species. In addition, the *Leucocytozoon* L_CIAE02 lineage was found in both adults and juveniles of the migratory black kite (*Milvus migrans*) in Tarifa (S Spain) [43], which suggests that this parasite lineage could be transmitted in both Africa and Europe. Interestingly, the two additional parasite lineages that we found in Eleonora’s falcons have previously been isolated from non-raptor species. *Haemoproteus* hBUBIBI01, which only differs in a single nucleotide from the lineage LK4, has been isolated for the first time from cattle egrets (*Bubulcus ibis*) in southern Spain [44]. Likewise, Illera *et al.* [45] have reported the presence of *Plasmodium* TF413, which is identical to lineage LK6, in Berthelot’s pipits (*Anthus berthelotii*), a resident species present in all the islands in the Canary archipelago. These latter authors [45] suggest that lesser kestrels, the only species previously found to be infected by the *Plasmodium* linage LK6, could have spread this lineage to Berthelot’s pipits. However, lesser kestrels do not breed in the Macaronesian islands and only vagrant individuals are ever observed in this area. In this respect and according to our results, a long-distant migratory raptor such as Eleonora’s falcon could spread blood parasites to resident birds on the main islands, where insect vectors are present (see [36]).

## Conclusions

Our results support the hypothesis proposed by Piersma [14] that explains the low prevalence of parasites in avian species living in marine environments and strongly indicates that in Eleonora’s falcons habitat characteristics affect the transmission of blood parasites.
